# Implementation of the Humanitude Care Methodology: contribution to the quality of health care*

**DOI:** 10.1590/1518-8345.2430-3123

**Published:** 2019-01-14

**Authors:** Liliana Vanessa Lúcio Henriques, Marilia de Assunção Rodrigues Ferreira Dourado, Rosa Cândida Carvalho Pereira de Melo, Luiza Hiromi Tanaka

**Affiliations:** 1 Santa Casa da Misericórdia, Coimbra, Portugal.; 2 Universidade de Coimbra, Faculdade de Medicina, Coimbra, Portugal.; 3 Escola Superior de Enfermagem de Coimbra, Coimbra, Portugal.; 4 Universidade Federal São Paulo, Escola Paulista de Enfermagem, São Paulo, SP, Brazil.

**Keywords:** Quality of Health Care, Humanization of Assistance, Patient-Centered Care, Nurse-Patient Relations, Old Age Assistance, Health Personnel, Qualidade da Assistência à Saúde, Humanização da Assistência, Assistência Centrada no Paciente, Relação Enfermeiro-Paciente, Assistência a Idosos, Pessoal de Saúde, Calidad de la Atención de Salud, Humanización de la Atención, Atención Dirigida al Paciente, Relaciones Enfermero-Paciente, Asistencia a los Ancianos, Personal de Salud

## Abstract

**Objective:**

to evaluate the contribution of the implementation of the Humanitude Care Methodology to the quality of health care in a Continuing Care Unit.

**Method:**

an action-research study with a non-probability convenience sampling, involving 34 health professionals from one unit in Portugal. Data was collected through a questionnaire and an observation worksheet for the Structured Sequence of Humanitude Care Procedures. We used data content analysis with the Statistical Package for Social Science, version 17.0.

**Results:**

health professionals demonstrated difficulties to provide care for people who are agitated, confused, disoriented, aggressive and who refuse care, and to communicate with patients who do not communicate verbally. The professionals valued the accomplishment of the stages of the observation worksheet. There were discrepancies between the perception of accomplishment and the actual practice. Throughout the implementation of the methodology, there was an increase in the practical application of the procedures, with positive repercussion for the patients and for the professionals.

**Conclusion:**

the results allowed to perceive the contribution of the process of implementation of the methodology, through the positive transformations in health care delivery.

## RESUMO

**Objetivo:**

avaliar a contribuição da implementação da Metodologia de Cuidado Humanitude para a qualidade da assistência à saúde em uma Unidade de Cuidados Continuados.

**Método:**

estudo de investigação-ação, que utilizou processo de amostragem não probabilística por conveniência, envolvendo 34 profissionais de saúde de uma unidade, em Portugal. A coleta de dados foi realizada por meio de questionário e planilha de observação de Sequência Estruturada de Procedimentos Cuidativos Humanitude. Utilizou-se análise de conteúdo dos dados e tratamento com Statistical Package for Social Science, versão 17.0.

**Resultados:**

os profissionais de saúde manifestaram dificuldades na prestação de cuidados de pessoas agitadas, confusas, desorientadas, agressivas e que recusavam os cuidados e, ainda, na comunicação com pacientes que não se expressavam verbalmente. Os profissionais valorizaram a realização das etapas da planilha de observação e verificaram discrepâncias entre a percepção de realização e a prática realmente efetivada. Ao longo da implementação da metodologia, observou-se aumento da aplicação prática dos procedimentos, repercutindo em ganhos para as pessoas cuidadas e para os profissionais.

**Conclusões:**

os resultados permitiram perceber a contribuição do processo de implementação da metodologia na transformação positiva da prestação de cuidados de saúde.

## RESUMEN

**Objetivo:**

evaluar la contribución de la implementación de la Metodología de Cuidado Humanitud para la calidad de la asistencia a la salud en una Unidad de Cuidados Continuos.

**Método:**

estudio de investigación y acción, mediante un proceso de muestreo no probabilístico por conveniencia, con la participación de 34 profesionales de la salud, desde una unidad, en Portugal. Recolección de datos realizada por medio de un cuestionario y una ficha de observación de Secuencia Estructurada de Procedimientos Cuidadativos Humanitud. Utilizado análisis de contenido de los datos y tratamiento con Statistical Package for Social Science, versión 17.0.

**Resultados:**

los profesionales de la salud manifestaron dificultades en la prestación de cuidados de personas agitadas, confusas, desorientadas, agresivas y que rechazan los cuidados y aún, de comunicarse con pacientes que no se comunican verbalmente. Los profesionales valoraron la realización de las etapas de la ficha de observación y verificaron discrepancias entre la percepción de realización y la práctica, realmente, efectiva. A lo largo de la implementación de la metodología, se observó un aumento de la aplicación práctica de los procedimientos, repercutiendo en beneficios para las personas cuidadas y para los profesionales.

**Conclusiones:**

los resultados permitieron percibir la contribución del proceso de implementación de la metodología, en la transformación positiva de la prestación de atención de salud.

## Introduction 

Global population ageing is one of the most important social phenomena since the mid-twentieth century. The significant demographic changes in Europe, with the increasing number of older people, have a social and economic impact^1^. Population ageing leads to an increase in the prevalence of major neurocognitive disorders (NCDs)^2^, commonly called “dementia” in literature^3^ ^-^ ^4^. Older adults with dementia commonly present signs of agitation and resistiveness to care. Health workers do not always know how to deal with these situations, which creates obstacles to the integration and rehabilitation of these people^5^. This reality is an invitation for caregivers to create new strategies that meet the needs of older adults with dementia. 

In 1979, Yves Gineste and Rosette Marescotti developed a care methodology called Gineste-Marescotti Care Methodology^®^ (MGM^®^) or Humanitude Care Methodology (HCM)^2^ ^,^ ^6^. Humanitude is the set of particularities that allow us to feel human species and recognize other human beings as the same species. The authors developed this care methodology based on concerns about dignity, freedom and autonomy in the daily care provided for dependent and vulnerable persons. 

The structured sequence of Humanitude care procedures is based on relationship pillars: gaze, speech, touch; and identity pillars: verticality. The caregiver provides an integrated care and, through positive sensory stimulation, enables the person to “live standing” and explore all their possibilities of living in relation to others^2^ ^,^ ^6^. 

This methodology starts a relationship through the Structured Sequence of Humanitude Care Procedures (SEPCH), with 5 consecutive and dynamic stages: openings, preliminaries, sensory circle, emotional consolidation and reunion. The openings are aimed at announcing the presence of the caregivers, opening relational channels, avoiding surprise approaches and respecting privacy and autonomy. The preliminaries represent the beginning of a relationship through the relationship pillars of Humanitude (gaze, speech and touch) and allow obtaining a relationship consent from the person receiving the care. The sensory circle includes the provision of care, with a consistent positive emotional environment between the caregiver and the patient using the Humanitude pillars. Emotional consolidation is a stage of cognitive and mental stimulation, which leaves a positive impression of the relationship and of the care in the emotional memory of the person receiving it, facilitating relationship consent and acceptance of future care^2^ ^,^ ^6^. The reunion is the final moment of the relationship, in which commitment to future care is affirmed. At this stage, farewells are said, and a new meeting is scheduled, preventing the feeling of abandonment^2^ ^,^ ^6^. 

In 2006, the National Network for Integrated Continuous Care (RNCCI) was created in Portugal for “people who are in a situation of dependency and need continuous health care and social support”^2^. Due to the diversity of patients, caregivers have been gradually recognizing the need to find innovative strategies to deliver quality care, appropriate to emerging needs^7^. In this context, caregivers of the RNCCI have been receiving training for the application of the HCM^2^, which has demonstrated effectiveness for approaching people with dementia or in situations of dependency, avoiding agitation and resistiveness to care^8^. 

The implementation of HCM^2^ ^,^ ^6^ occurs in four stages: awareness - 15hrs of theoretical and practical training involving formal and informal leaders; dissemination - 35hrs of training and actions in the context of care, lasting 5 days and involving technical direction and caregivers, providing training on the Humanitude care procedures; consolidation - 7hrs training, directed to the ‘support group’, the strategic management team responsible for monitoring and following the implementation of the HCM^2^. This Care Methodology^2^ promotes care based on rules such as: never abandoning care, never surprising the person with the approach and respecting the household of the patient, following the SEPCH^2^ ^).^ The present study was carried out with the objective of evaluating the contribution of the implementation of the Humanitude Care Methodology for the quality of care in a Continuing Care Unit in Portugal. To that end, the situation was evaluated to verify if there were differences between the importance attributed to each SEPCH step, to assess the perception of accomplishment and to observe the actual practice of health professionals. Afterwards, the transformations in the care provided throughout the implementation process were monitored. 

## Method 

This is a longitudinal action research study using a model with sequential, interdependent and interrelated stages^2^. A non-probability convenience sampling was used, with the participation of 34 health professionals from the Continuous Care Unit (CCU) who provided direct care for older adults. The sample included 18 professionals who had already completed the training in Humanitude and 16 professionals who had not completed the training and were learning by observing the practice of colleagues. The Unit was chosen because it was in the initial phase of the implementation of the HCM^2^. None of the professionals refused to participate and there were no dropouts during the study. The participants were made aware that the objective of the study was to follow the implementation process of the HCM^2^ in the CCU. 

This Health Unit provides care aimed at preventing or slowing down situations of dependency, improving the patients’ health status as much as possible in a period of hospitalization of 90 days. It has 30 hospitalization beds and most of the patients are dependent and present cognitive impairment. The study was conducted during four months, between September and December 2016.

Data was collected by the researcher in three stages: in the first stage, the 34 professionals answered a questionnaire with the following socio-demographic variables: age, gender, profession and experience in the profession. It also assessed the participants’ experiences in providing care to older adults and the difficulties felt in this care. The form was delivered by the researcher in the Health Unit, in a sealed envelope addressed to each professional. Afterwards, they were collected by the Directors of the CCU to return to the researchers. The instrument SEPCH, constructed and validated in Portugal^2^, was used to verify the importance attributed to each Humanitude care procedure and the perception of accomplishment of the care procedures. The SEPCH consists of 31 items, each one representing a Humanitude care procedure. It evaluates the importance attributed by each professional to each item, through a 4-point Likert scale, in which 1 corresponds to “Without importance” and 4 to “Extremely important”. The SEPCH also evaluates the perception of accomplishment of each Humanitude care procedure through a 4-point Likert scale in which 1 corresponds to “I never do this” and 4 “I always do this”. The second stage of data collection was conducted with 18 professionals who had undergone HCM training^2^ and answered the following open questions: “How does HCM^2^ ^)^ facilitate care delivery?” and “What is your perception regarding the contribution of the implementation of HCM^2^ for patients?” The third stage evaluated the coherence between the perception of accomplishment of each Humanitude care procedure and its actual practice. The measurement was done through the SEPCH worksheet. The participant observation was conducted by two observers with knowledge and experience in the application of this observation sheet. Observation occurred during care activities of hygiene, comfort, feeding and mobilization, in 2 consecutive days, in the beginning of each month, during 4 months, from September to December 2016. After identifying the Humanitude care procedures which were less consistent and required improvements, some strategies began to be implemented: training in action, flyers, placement of labels and autoscopy of the care for later analysis and reflection with the professionals, in order to raise awareness^9^ ^-^ ^10^. The objective of these strategies was to improve the use of HCM^2^ and the quality of care. 

Quantitative data analysis was performed using the Statistical Package for Social Sciences version 17.0. Descriptive statistics were used to analyze the characterization of the participants and the identification of difficulties in the care of older adults. The Friedman test was used to evaluate the differences between the four moments of observation, with a level of significance of 0.05. The technique of content analysis according to Bardin was used for the qualitative data^11^. In the pre-analysis stage the transcriptions were read and re-read, highlighting the importance of some set of elements within the universe of the documents analyzed. In the exploratory phase, data was coded, classified and categorized by two researchers, leading to the end of the analysis process with the interpretations and inferences about the qualitative data. The themes identified were derived from the data obtained.

The triangulation of the qualitative and quantitative data was done through analysis and reflection on the main difficulties of the professionals in the practice of care, the inconsistencies between the perception of accomplishment of the Humanitude care procedures and their application in practice and how the use of the HCM^2^ improved the provision of care and the contribution of the patients. 

This study was approved by the Research Ethics Committee of the School of Medicine of the University of Coimbra, on July 14, 2016, with the registration number 056/2016. The study obtained authorization from the coordination of the institution where the data were collected. After explaining the objectives of the study and the voluntary option to participate, clarifying that acceptance or refusal would not cause any harm to the participants, the informed consent form was signed.

## Results

Among the 34 professionals, most were females and the mean age was 36 years. Participants came from nine distinct professional areas, and the most representative were medical assistants (11) and nurses (11). The sample also included 1 sociocultural animator, 1 social worker, 1 physician, 1 psychologist, 1 speech therapist and 1 general coordinator. The mean time of experience of the professionals was 5 years and 55.9% worked in the current area and functions for between 6 months and a year and a half. 

Of the 34 professionals participating in the study, all had provided or currently provided care for older adults; 32 provided care for people with sequelae of stroke, 33 for people with dementia and 31 for people with aggressive emotional states and agitation. 

When questioned about the main difficulties experienced in this care, 17 reported difficulties in caring for people who were agitated, confused, disoriented, aggressive and who refused care; 15 participants reported having difficulty communicating with patients who did not communicate verbally; 12 expressed difficulties in providing care for terminally ill patients; and four responded that they had difficulty in providing hygiene care. 

Of the 34 participants in the study, 18 had undergone training in HCM^2^. Of these, 100% considered that Humanitude care procedures contributed to reduce difficulties and facilitate care delivery. 

Three categories emerged from the question: “How does HCM^2^ facilitate care delivery?”: operationalization of the relationship; greater awareness of the potential of the person; and better understanding of the person who receives care. According to the participants, the operationalization of the relationship is accomplished by learning relational techniques that facilitate the relationship, as reported: *In some situations, patients collaborate and feel more relaxed when we explain the procedures, and thus collaborate and let us help them* (P4) and by learning verticalization techniques, as reported by this professional: *Easier to position and transfer patients with less mobility* (P10). Regarding the awareness of the potential of the person, the professionals highlighted the valuation of the potentialities of the person: *These procedures value the patients I take care of (…)* (P5), the appropriate duration of care, as reported by this professional: *I also learned that we should give some patients more time so they can achieve greater participation in certain activities* (P1) and greater intentionality in the relationship with the person receiving care, as stated by this professional: *the training was useful because I am more careful about the way I speak, and about the importance of the gaze and the touch* (P1). 

**Figure 1:**
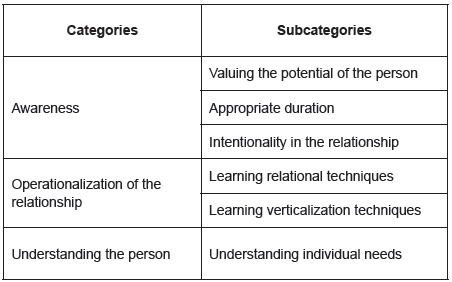
Perception of how the Humanitude Care Methodology facilitates care delivery, Leiria, PT, Portugal, 2016

Figure 1 presents the categories and subcategories of the professionals’ perception about how the Humanitude Care Methodology facilitates care delivery.

Four categories emerged from the question: “What is the contribution of the implementation of HCM^2^ for patients?”: reduction of agitation, resistiveness and refusal of care; increased communication; reduction of physical and mental degradation; and improvement of the quality of life of patients. Regarding the reduction of agitation, resistiveness and refusal of care, professionals have more often mentioned calmness and tranquility, which are present in the following speech: *It was good because I learned to better deal with them and to make them calmer* (P11). They also report a decrease in agitation, as demonstrated by this professional: *Decreased agitation during meals and decreased complaints in decubitus change* (P9). They show greater receptivity and acceptance of care; and greater collaboration and involvement in care, as reported by this professional: *The impact was positive as some of the older adults became more involved and cooperative during care* (P17). Regarding the category increased communication, the promotion of interaction was evidenced, as reported by this professional: *Humanitude training increases interaction with patients (…)* (P1). Regarding the improvement of communication between professionals and patients, one participant informed that: *(…) communication with the elderly has become easier* (P16). The following subcategories emerged from the category reduction of physical and mental degradation: more active patients, as demonstrated in these speeches: *(…) The impact was very positive as some older people became more reactive* (P17), *there was more participation in certain activities* (P1); increased verticality, as reported by this professional: *I started to lift the patients from the bed and take them to the bathroom to provide hygiene care (…)* (P6); and increased autonomy, as in the speech: *we observe the patients making decisions regarding care and achieving more autonomy* (P18). In the category improvement of the quality of life of the patients, this professional reported that: *the fact of making patients happier and with better quality of life* (P16), and patients feel more valued: *(…) these procedures value the patients I take care of (…)* (P5). It also made the patients feel useful, as mentioned by this professional: *(…) allows the patient to feel useful* (P13), another professional emphasizes dignity: *(…) increases the dignity of the patient(…) relieves pain and increases privacy* (P5), one professional refers to increased comfort: *(…) and promotes more comfort for patients* (P3). 

**Figure 2:**
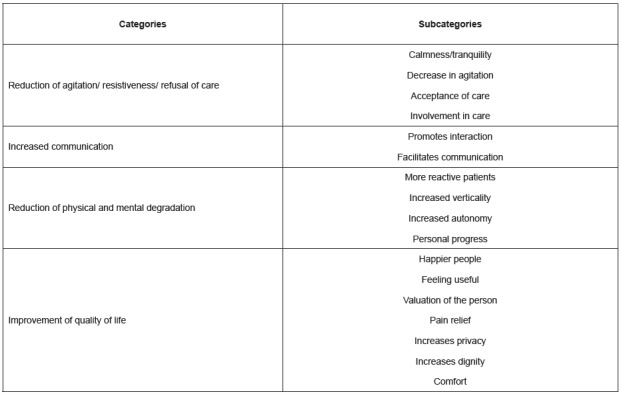
Perception of the professionals about the contribution of the Humanitude Care Methodology for the patient, Leiria, PT, Portugal 2016

Figure 2 shows the categories and subcategories of the professionals’ perception about the contribution of the Humanitude Care Methodology^2^ for the patient.

In the analysis of the results obtained in the application of the SEPCH questionnaire to the 34 professionals who evaluated the Humanitude care procedures structured in 5 stages, we verified that the professionals assigned great importance to the SEPCH stages, reaching an overall score of 83.8%. Openings were evaluated with a total score of 80.7%, preliminaries, with 82.19%, sensory circle, with 86.21%, emotional consolidation, with 86.27% and the reunion with 80.64%. 

Regarding the perception of the professionals about their practice of each of the SEPCH items, we found that they believed they consistently applied the SEPCH items, reaching an overall score of 74.48%. The total score was 72.43% in the openings, 72.43% in the preliminaries, 75.92% in the sensory circle, 76.23% in the emotional consolidation and 75.25% in the reunion. The data obtained show that the professionals attribute higher scores to the importance of the SEPCH stages than to the perception of accomplishing them in practice. 

In the first observation of the care provided by the professionals using the SEPCH observation worksheet, we obtained an overall score of 52.16%. The openings obtained a score of 43.75%, with items varying from 18.18% to 84.09%. Preliminaries reached a total score of 65.40%, with scores distributed between 2.27% and 90.91%. The sensory circle presented a total score of 52.27%, with items distributed between 27.27% and 77.27%. The emotional consolidation presented a total score of 30.30%, with scores varying between 13.64% and 45.45%. The reunion had a total score of 42.42%, with scores distributed between 22.73% and 63.64%.

The general analysis of the results obtained in the comparison between the importance attributed to the SEPCH items, the perception of accomplishment and the first observation showed a percentage decrease in all stages. The greatest discrepancy was in emotional consolidation, with a difference of 45.93% between the perception of accomplishment and the observation of the practice. The smallest discrepancy between the perception of accomplishment and the actual practice was in the preliminaries, with 7.23%. As a result of the application of the SEPCH questionnaire to professionals, the comparison between the importance attributed, the perception of accomplishment and the results of the first observation can be seen in Table 1. 

**Table 1:** Importance attributed by professionals to the stages of the Structured Sequence of Humanitude Care Procedures. Comparison with the perception of accomplishment and the first practical observation, Leiria, PT, Portugal, 2016

Stages of the SEPCH*	Score %		
	Importance attributed	Perception of accomplishment	First observation
Total openings	80.70	72.43	43.75
Total preliminaries	82.19	72.63	66.29
Total sensory circle	86.21	75.92	52.27
Total emotional consolidation	86.27	76.23	30.30
Total reunion	80.64	75.25	42.42
Overall total	83.80	74.48	52.16

* SEPCH - Structured Sequence of Humanitude Care Procedures

The second observation, carried out the following month, had an overall score of 63.49%. The total score in openings was 71.59%, with items’ scores distributed from 40.91% to 90.91%. The preliminaries reached a total score of 77.02%, with scores between 18.18% and 100%. The sensory circle presented a total score of 62.12%, with scores varying between 45.45% and 95.45%. The emotional consolidation dimension presented a total score of 29.55%, with scores distributed between 18.18% and 40.91%. The reunion had a total score of 51.52%, with scores varying between 13.64% and 90.91%. 

The third observation had an overall score of 72.95%. The openings had a total score of 71.59%, with items’ scores distributed from 45.45% to 95.45%. The preliminaries reached a total score of 83.59%, with items’ scores varying between 34.09% and 100%. The sensory circle had a total score of 76.52%, with scores distributed between 50% and 100%. The emotional consolidation presented a total score of 37.12%, with scores distributed between 11.36% and 72.73%. The reunion dimension had a total score of 64.39%, with items distributed between 27.27% and 88.64%.

The fourth observation showed an overall score of 78.23%. The openings had a total score of 82.39%, with items distributed from 70.45% to 97.73%. The preliminaries reached a total score of 85.86%, with scores varying between 31.82% and 100%. The sensory circle presented a total score of 81.06%, with scores distributed between 45.45% and 100%. The emotional consolidation had a total score of 41.67%, with scores varying between 15.91% and 75%. The reunion demonstrated a total score of 75%, with items distributed between 52.27% and 88.64%. In Table 2 we can observe the data obtained in the four moments of observation. 

**Table 2:** Comparison of the stages of the Structured Sequence of Humanitude Care Procedures in the four moments of observation in percentage, Leiria, PT, Portugal, 2016

Stages of the SEPCH*	**Observations %**			
	**First**	**Second**	**Third**	**Fourth**
**Total openings**	**43.75**	**71.59**	**71.59**	**82.39**
**Total preliminaries**	**66.29**	**77.02**	**83.59**	**85.86**
**Total sensory circle**	**52.27**	**62.12**	**76.52**	**81.06**
**Total emotional consolidation**	**30.30**	**29.55**	**37.12**	**41.67**
**Total reunion**	**42.42**	**51.52**	**64.39**	**75**
**Overall total**	**52.16**	**63.49**	**72.95**	**78.23**

* SEPCH - Structured Sequence of Humanitude Care Procedures

The four observations demonstrated that the items of the SEPCH with lower values ​​were: item “7. Introduce themselves to the patient (e.g.: I am…)”, item “3. If there is no answer (expressed or implied), enters the room quietly and knocks on the bed bar” and item “26. Tells the patients about the pleasant experience of being with them (e.g.: I enjoyed knowing you, being with you).” These items require more investment in training. The items with higher values ​​were item “6. Call the patients by the name that they prefer”, item “9. Uses a calm, melodic and soft tone of voice”, item “8. Tells the person the reason for the encounter (e.g.: there to be with them, to talk, to help)”, item “21. Uses a calm and smooth tone of voice during care”, and item “22. Frequently repeats the person’s name”. All these items were observed in 100% of the observations in the fourth round. The promotion of verticality (item 24 of the SEPCH) was one of the items that was improved, showing an increase between the first and fourth observations, going from 52.27% in the first observation to 79.55% in the fourth observation. 

The analysis of the SEPCH stages (Openings, Preliminaries, Sensory Circle, Emotional Consolidation and Reunion) in the 4 moments of observation showed an increase in the means of all stages, except emotional consolidation, which presented a slightly decrease in the second observation. We verified a higher mean in Sensory Circle and Preliminaries and lower means in Emotional Consolidation and Reunion in the 4 moments. 

Using the Friedman Test to evaluate the differences in the 4 moments of observations for each stage of the SEPCH, we found a statistically significant difference in the Openings (**χ** ^**2**^ =12.903; p=.005), the Preliminaries (**χ** ^**2**^ =15.104; p=.002), the Sensory Circle (**χ** ^**2**^ =23.800; p=.000) and the Reunion (**χ** ^**2**^ =11.326; p=.010). In the Emotional Consolidation stage there was no statistically significant difference (**χ** ^**2**^ =2.578; p=/461). In Table 3 we can see the means and standard deviations of the SEPCH stages in the 4 observations of the study. 

**Table 3:** Mean and Standard Deviaton of the stages of the Structured Sequence of Humanitude Care Procedures in the four observations of the study, Leiria, PT, Portugal, 2016

Stages of the SEPCH*	1^st^ observation		2^nd^ observation		3^rd^ observation		4^th^ observation	
	Mean	SD^†^	Mean	SD^†^	Mean	SD^†^	Mean	SD^†^
Openings	1.75	1.18	2.86	1.32	2.86	1.19	3.29	1.11
Preliminaries	5.89	1.93	6.93	1.61	7.52	0.84	7.73	0.94
Sensory Circle	6.27	2.78	7.45	2.61	9.14	1.49	9.75	1.42
Emotional Consolidation	0.91	0.92	0.89	1.07	1.11	0.92	1.25	0.86
Reunion	1.32	1.08	1.55	0.80	1.90	0.88	2.25	0.87

* SEPCH - Structured Sequence of Humanitude Care Procedures; †SD - Standard Deviation

## Discussion

The participants of the study had different professional backgrounds, but all of them directly related to the direct provision of health care. In addition to being a young team, more than half of the participants had been working in the current area and function for between half a year and a year and a half, meaning that most professionals were still beginners and required follow-up, training, reflection on practices and studying^12^. 

Despite the variations in time providing direct care to older adults, patients with dementia or with sequelae of stroke and people with aggressive emotional states/agitation behaviors, all the professionals participating in the study had already provided care to people with these clinical conditions. The difficulties most reported by the professionals were providing care for people who are agitated, confused, disoriented, aggressive and who refuse care. These data were corroborated in another study^13^, in which caregivers expressed difficulties in providing care for people with behavioral, cognitive and emotional alterations Participants also expressed difficulty communicating with people who did not communicate verbally. This was also identified in another study^13^, due to the difficulty in understanding non-verbal communication and the lack of professionalization of speech^2^. 

Difficulty providing care to terminally ill patients was reported by the professionals of our study, similar results to those obtained in a study carried out with health professionals^14^. 

Despite its smaller percentage, the provision of hygiene care was also pointed as a difficulty. These results may be related to the use of techniques that are not adequate to the needs of the patients and to the reality of care, leading to high levels of physical and emotional discomfort among people with dementia and increasing behavioral and psychological symptoms of dementia, as evidenced in recent studies^2^ ^,^ ^8^ ^,^ ^15^ ^-^ ^16^. 

All the professionals who underwent training in the HCM^2^ considered that Humanitude care procedures contributed to reduce the difficulties in providing care, facilitating care delivery. In a study carried out with health professionals^7^, the participants also recognized the important contributions of HCM training, highlighting the reduction of resistiveness to care, reduction of agitation, reduction of problems related to immobility, promotion of autonomy and respect for the dignity of the human person. Participants reported that they had overcome their communication difficulties through the application of the HCM^2^ ^,^ ^8^. Considering these results, it is fundamental to train professionals on innovative care methodologies appropriate to the reality of care, with care procedures that operationalize and systematize the relationship^6^. The HCM^2^, with benefits that are scientifically validated in national and international scenarios, seems to be an innovative care tool that allows professionals to be trained with relational techniques that professionalize the relationship between the caregiver and the patient reducing the difficulties in providing care^2^.

The answers obtained from the professionals demonstrate a good acceptance of the SEPCH stages, given the great importance attributed to these stages. In addition to these data, the data related to the perception of accomplishment of the steps also maintains high values, despite the percentage decreases when compared to the importance attributed. Similar results were obtained in a study^2^ in which the total score of the importance attributed to the items was higher than the overall perception of accomplishment of SEPCH stages. In this study, nurses attributed great importance to the Humanitude care procedures and had a high perception of their application in practice. 

However, there was a discrepancy between the perception of accomplishment and the actual practice, during observations. The first observation allowed us to perceive that there is a difference between the perception of accomplishment and the practice carried out in all the SEPCH stages. In this first observation the professionals demonstrated difficulty in complying with SEPCH procedures. A study conducted with nurses from UCC also presented similar results^2^, with differences between the importance attributed by professionals and the actual practice. 

Despite the gradual increases in actual practice throughout the observations, after the implementation of several strategies to facilitate the use of the HCM^2^, it was verified that emotional consolidation was the stage where the professionals presented greater difficulties for implementing the care procedures. Another study^2^ also identified difficulties to value the encounter with positive words and tender gestures, producing a positive priming in the emotional memory of the person. 

There was a positive evolution between the first and fourth observations in all dimensions of the SEPCH. This may be related to the interventions carried out during the HCM implementation process^2^, during which the professionals integrated the cares in their practice. The stage that showed the greatest positive evolution between the first and fourth observations were the openings.

The increase in verticality may be related to the encouragement to walk, reducing the number of people who use a wheelchair and who remain in bed, and encouraging the performance of hygiene procedures standing in the bathroom. The mean duration of hygiene care decreased throughout the observations, and hygiene care began to be completely carried out in the bathroom, reducing pressure injuries in patients. 

The professionals identified a reduction of physical and mental degradation as a relevant point in the application of this methodology. For the participants, this methodology contributed to the improvement of quality of life, which is in agreement with the results of other studies developed in hospital environments, which verified that the application of this care methodology is a valuable tool for the control of agitation in older adults with dementia^8^, for the reduction in medication use^8^ and consequently, the reduction of risks associated.

These results may be reflective of the effects of a non-pharmacological, low-cost measure on the care provided to dependent, vulnerable and dementia patients. Exploring this methodology in academic and professional contexts can have important consequences on management, organization, education and health of the institutions^2^.

The main limitation of the present study was the size of the sample, since the low number of participants does not allow the generalization of results. It is necessary to conduct more research on the effectiveness of this care methodology from the perspective of family members. Also, it is necessary to increase, along with health professionals, the contribution to the implementation and change of culture of care and public health policies, especially in the context of an aging population with specific characteristics and who require specialized care, aimed at transforming routine care into conscientious care.

## Conclusion 

HCM training facilitated care delivery and the professional perceived positive feelings among patients when applying knowledge in practice. 

The professionals attribute great importance to the SEPCH stages and perceive the accomplishment of Humanitude care procedures. However, the perception of accomplishment of the care procedures is higher than the actual practice. 

The implementation of the HCM had results in the change of practices of the caregivers. The development of this study allowed to verify that, despite the importance attributed to this methodology by the professionals, there is, in fact, a discrepancy between what they perceive they are doing and what they actually do. With the development of specific interventions and training in action, it was possible to verify that, throughout the several observations, there was a gradual reduction of that difference, with a gradual and consistent increase in the execution of the Humanitude care procedures. Some procedures were internalized in such a way that they reached maximum values in the observation. However, there were others, corresponding to the emotional consolidation, in which health professionals demonstrated greater difficulty of integration and application in practice. There was a need for following and monitoring the implementation process, for the sustainability and consolidation of good practices by all professionals, contributing to the transformation and quality of health care. 
